# When plant virology met *Agrobacterium*: the rise of the deconstructed clones

**DOI:** 10.1111/pbi.12412

**Published:** 2015-06-12

**Authors:** Hadrien Peyret, George P. Lomonossoff

**Affiliations:** ^1^ Department of Biological ChemistryJohn Innes CentreNorwich Research ParkNorwichUK

**Keywords:** molecular farming, plant virus vectors, *Agrobacterium*, deconstructed viral vectors, agroinfiltration, recombinant protein expression in plants

## Abstract

In the early days of molecular farming, *Agrobacterium*‐mediated stable genetic transformation and the use of plant virus‐based vectors were considered separate and competing technologies with complementary strengths and weaknesses. The demonstration that ‘agroinfection’ was the most efficient way of delivering virus‐based vectors to their target plants blurred the distinction between the two technologies and permitted the development of ‘deconstructed’ vectors based on a number of plant viruses. The tobamoviruses, potexviruses, tobraviruses, geminiviruses and comoviruses have all been shown to be particularly well suited to the development of such vectors in dicotyledonous plants, while the development of equivalent vectors for use in monocotyledonous plants has lagged behind. Deconstructed viral vectors have proved extremely effective at the rapid, high‐level production of a number of pharmaceutical proteins, some of which are currently undergoing clinical evaluation.

## Introduction

The use of plants as expression systems for the production of recombinant proteins has emerged as an attractive alternative to systems based on bacteria, yeast or animal cells. Producing proteins in plants potentially has advantages in terms of cost as well as safety, as plant pathogens do not infect mammals, and mammalian pathogens do not infect plants, thus reducing contamination risks when pharmaceutical proteins are produced (Kusnadi *et al*., 1997; Lico *et al*., 2008). Moreover, certain plants such as *Nicotiana benthamiana* can be grown in high density and still produce large amounts of biomass in a matter of weeks. Historically, there have been essentially two approaches to producing heterologous proteins in plants—stable transformation (either nuclear or plastid) and transient expression. Stable transformation involves the production of true‐breeding lines of genetically transformed plants. This approach has advantages in terms of reproducibility and potential large‐scale production; however, it is often very time‐consuming and is unsuitable for the rapid screening of a wide variety of different constructs (Kusnadi *et al*., 1997). Transient expression in plants allows for the production of recombinant proteins in a matter of days in what is essentially a batch process (Pogue *et al*., 2002). Furthermore, processes have been developed for scaling‐up production of transiently expressed proteins in plants (D'Aoust *et al*., 2010), enabling this approach to compete with the large amount of material that can be produced through stable transformation.

This history of plant molecular farming dates back to the 1970s, when it was discovered that the Gram‐negative bacterium *Agrobacterium tumefaciens* contains a special plasmid, named Ti (tumour‐inducing), which is capable of directing the transfer of part of the plasmid (the transfer DNA, or T‐DNA), to the plant cell, and integrating the T‐DNA into the plant genome (Chilton *et al*., 1977). At around the same time, it was proposed that the plant virus, cauliflower mosaic virus (CaMV) which has a circular double‐stranded DNA (dsDNA) genome, could be used for the episomal expression of foreign proteins in plants in a manner analogous to bacterial plasmids or vectors based on the mammalian dsDNA virus, simian vacuolating virus 40 (SV40; Hull, 1978; Szeto *et al*., 1977). This early work led to rapid advances in the fields of both plant transformation and the development of plant virus‐based vectors for transient expression. This review will discuss how the two approaches, which were initially considered distinct, have merged in recent years and led to the deployment of deconstructed viral vector systems.

## Development of methods for *Agrobacterium*‐based transformation

In the case of stable transformation, research initially focussed on controlling the natural transformation process *in vitro*, by infecting tobacco cells (Marton *et al*., 1979) or protoplasts (Wullems *et al*., 1981a) with *A. tumefaciens* and regenerating genetically modified shoots from these cells. At the time, the only way to obtain complete plants from such modifications was to graft regenerated shoots onto healthy tobacco plant root stock, and this allowed the plant to flower, set seed and produce transgenic offspring (Wullems *et al*., 1981b). However, it was quickly shown that disabling the tumour‐controlling genes of the Ti plasmid allowed plants to be regenerated directly after transformation with *A. tumefaciens* (Barton *et al*., 1983; Greve *et al*., 1982). Such mutated varieties of *Agrobacterium* were called ‘disarmed strains’. Up to this point, plants were only transformed with a native *A. tumefaciens* plasmid, so the introduced transgenes were restricted to bacterial opine synthesis and tumour‐inducing genes.

The leap from research to biotechnology was made in 1983. Heterologous genes were inserted into the T‐DNA: yeast alcohol dehydrogenase and bacterial neomycin phosphotransferase (which confers kanamycin resistance) were transferred to plants alongside the native nopaline synthase (*nos*) gene. While the genes were shown to be present, only nopaline (the product of the *nos* gene) was shown to be produced (Barton *et al*., 1983). However, it was very quickly realized that this could be a promoter issue, so antibiotic resistance genes were placed downstream of the *nos* gene promoter, allowing the expression of antibiotic resistance genes in transgenic plants (Herrera‐Estrella *et al*., 1983). Another significant breakthrough came when it was discovered that the *vir* (virulence) genes on the Ti plasmid which direct the transfer of the T‐DNA into the plant cell and its integration into the plant genome could be dissociated from the T‐DNA itself. That is to say, the *vir* genes could be placed on one plasmid which could act as genetic background, while the T‐DNA could be placed on a separate small plasmid that would be easy to manipulate in *Escherichia coli*. The T‐DNA on this ‘binary vector’, as it became known, could be modified to contain a selectable marker (such as a kanamycin resistance gene) and a multiple cloning site for the insertion of heterologous genes of interest, or GOIs (Bevan, 1984; de Framond *et al*., 1983; Hoekema *et al*., 1983).

These binary vectors have been continually improved as a result of new research into the T‐DNA transfer process. For example, it was discovered that it was more effective to place the GOI near the right border (RB) of the T‐DNA and the selection marker near the left border (LB), because the RB is the first to be integrated into the plant genome (Wang *et al*., 1984) and because there can sometimes be incomplete transfer of the T‐DNA at the LB (Rossi *et al*., 1996). This ensures that transformants displaying antibiotic resistance will also contain the GOI. This paradigm shift in biotechnology did not go unnoticed by industry: indeed, one of the first binary vectors was created by a team from Monsanto (Fraley *et al*., 1983), who considered that the field was now wide open for any gene, from any species, to be expressed in plants. The primary result of this was the rapid development of industrial‐scale genetic engineering technology for agricultural purposes (Marshall, 2012), but at the same time it was also the birth of plant molecular farming (Hiatt *et al*., 1989).

The next improvement was agroinfiltration to directly express proteins: this involved the use of negative pressure to flood the intercellular space of a plant leaf with *Agrobacterium* (vacuum infiltration), as opposed to simply inoculating a small spot on a leaf. This technique was actually first described for the purpose of ‘floral dipping’ of whole adult *Arabidopsis* plants to generate stable transformants (Bechtold and Pelletier, 1998; Bechtold *et al*., 1993; Clough and Bent, 1998). However, research groups wanting to transiently express proteins of interest preferred to vacuum‐infiltrate the excised leaves of bean and tobacco plants (Kapila *et al*., 1997; Vaquero *et al*., 1999). Schob *et al*. (1997) then described a variant of the technique known as ‘syringe infiltration’, in which a needle‐less syringe is used to agroinfiltrate the nonexcised leaves of whole plants, without the need for a vacuum chamber. The first description of vacuum infiltration of whole plants, and not just excised leaves, for transient expression purposes, was not published until almost a decade later (Marillonnet *et al*., 2005), with detailed methods described after that (Garabagi *et al*., 2012; Gleba *et al*., 2007).

## The development of plant virus‐based expression vectors

Although CaMV had been proposed as an expression vector in the late 1970s (Hull, 1978; Szeto *et al*., 1977), it was not until the demonstration that cloned copies of the viral DNA were infectious when mechanically inoculated on plants (Howell *et al*., 1980) that vector design could begin in earnest. This involved identifying potential sites for the insertion of foreign genes where their presence would not adversely affect virus replication. Unfortunately, the replication mechanism of CaMV was more complex than originally envisaged and, unlike that of bacterial plasmids or SV40, involves an RNA intermediate (Pfeiffer and Hohn, 1983). As a result, the virus has a very high recombination rate leading to rapid loss of inserted sequences. For reviews of this early work on CaMV‐based vectors, see Porta and Lomonossoff (1996, 2002). Consequently, although CaMV‐based vectors have not proved particularly useful for protein expression in plants, components from the virus, most notably the ubiquitous CaMV 35S promoter, have played an essential role in the development of plant biotechnology (Pietrzak *et al*., 1986).

Subsequent to the work on CaMV, attention turned to the development of other viruses as potential gene vectors, including those with single‐stranded DNA (ssDNA) and ssRNA genomes. While vectors based on the ssDNA‐containing geminiviruses could, in many cases, be delivered by mechanical inoculation of cloned dsDNA copies, the delivery of vectors based on ssRNA viruses initially required an *in vitro* transcription step to copy a dsDNA cDNA clone of the genome into infectious RNA (Ahlquist and Janda, 1984). The need for this transcription step was obviated by the demonstration that cloned cDNA copies of an RNA genome are infectious if the virus‐specific sequence is positioned downstream of a strong plant promoter, in this case the CaMV 35S promoter (Mori *et al*., 1991). This was considered somewhat surprising as primary RNA transcribed from cDNA in the nucleus would have different 5′ and 3′ sequences compared to viral RNA copied by the viral replicase.

## The great merger: combining plant viruses with *Agrobacterium*

The requirement to mechanically inoculate nucleic acid onto plants had two effects on the early development of plant virus‐based vectors. First, it restricted vector development to those viruses which are mechanically transmissible, and second, it was highly inefficient, introducing the infectious sequence to only a very few cells on the inoculated leaf. Thus, to achieve high levels of expression in leaf tissue, it was vital to ensure that the introduced viral sequence retained its ability to spread from the initially infected cell, a fact which had consequences for vector design. The solution to this limitation was found by utilizing the ability of *Agrobacterium* to efficiently deliver DNA to plant cells. This is the point at which the nascent field of plant transformation fully merged with plant virology: when it was shown that inserting tandem copies of the CaMV genome into a T‐DNA of a binary vector allowed a viral infection to be initiated on a mature plant via *Agrobacterium‐*mediated gene transfer (Grimsley *et al*., 1986). This technique, called ‘agroinfection’ and later ‘agroinoculation’, involved pipetting a small volume of a suspension of modified bacteria onto an abraded plant leaf. From there, the bacteria are able to infect the plant with the virus, which then spreads systemically. This technique was subsequently used to infect maize plants with cloned copies of a geminivirus, maize streak virus (MSV); this was a breakthrough in the development of MSV as a vector because the virus is not mechanically transmissible (Grimsley *et al*., 1987). This report was also significant as it showed that *A. tumefaciens* could deliver sequences to monocots, a group of plants that the bacterium does not normally infect. The first report of the application of agroinfection to an RNA virus was by Leiser *et al*. (1992), who showed that cloned copies of beet western yellows virus, which were not infectious as naked DNA, could initiate an infection when introduced via agroinfection.

The subsequent invention of agroinfiltration quickly made *Agrobacterium* the tool of choice for initiating infections with virus‐based vectors. In addition, the ability to simultaneously deliver T‐DNA to the majority of cells in a leaf meant that it was no longer essential that the virus should be able to spread out from the initially infected cells in order to achieve high levels of expression. This had a major consequence in terms of vector development: it allowed the creation of ‘deconstructed’ viral vectors. This involved removing viral genes that were not strictly necessary to the production of the recombinant protein, such as those coding for the viral coat protein (CP) or movement proteins (MP), and replacing these genes with one or more GOIs. This evolution has taken place with all of the most popular viruses used as the basis of expression vectors: tobamoviruses, potexviruses, tobraviruses, geminiviruses and comoviruses.

## Tobamovirus‐based vectors

**Figure 1:**
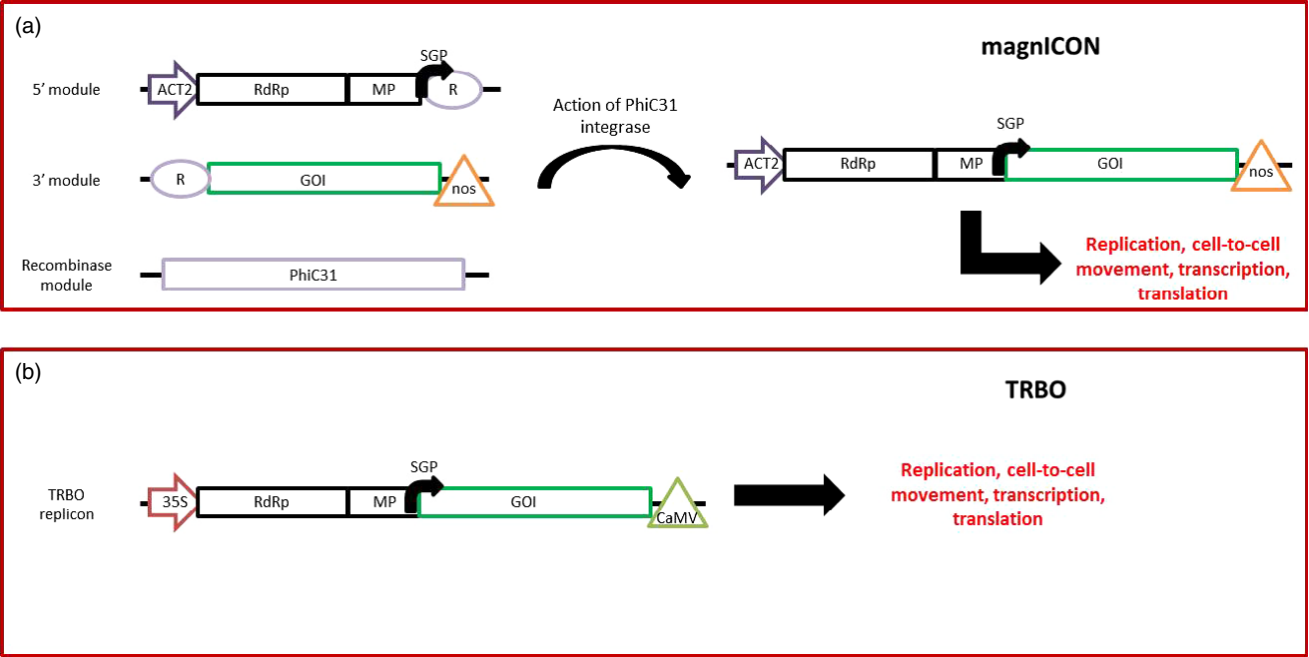
Simplified diagrams of deconstructed tobamovirus‐based expression systems. (a) The magnICON system (Marillonnet *et al*., 2004) is composed of three modules. The recombinase module allows the recombination of the 5′ and 3′ modules to form a complete replicon. ACT2: Arabidopsis actin 2 promoter, RdRp: tobamovirus RNA‐dependent RNA polymerase, MP: tobamovirus movement protein, SGP: TMV subgenomic promoter directing transcription of the gene of interest (GOI), R: recombination site, nos: nos terminator, PhiC31: streptomyces phage 31 integrase. (b) The TRBO system (Lindbo, 2007) is composed of a single module. 35S: 35S promoter, CaMV: cauliflower mosaic virus terminator, other abbreviations as above. Both replicons are capable of replication, cell‐to‐cell (but not systemic) movement, transcription and translation.

Tobamoviruses, of which tobacco mosaic virus (TMV) is the type species, are rigid rod‐shaped single‐stranded positive‐sense RNA (+ssRNA) viruses. Probably the most famous example of a deconstructed viral vector is the magnICON system (Figure 1a), in which a hybrid tobamovirus genome with elements from TMV and turnip vein‐clearing virus (TVCV) is split into three components which are co‐infiltrated into the same plant's leaves from a mix of three *Agrobacterium* cultures (Marillonnet *et al*., 2004). While this was certainly not the first tobamovirus‐based vector to be used for the expression of proteins in plants (Dawson *et al*., 1989; Donson *et al*., 1991; Hamamoto *et al*., 1993; Shivprasad *et al*., 1999; Sugiyama *et al*., 1995; Takamatsu *et al*., 1987; Turpen *et al*., 1995), it is the first to be rationally deconstructed with high yield of recombinant protein as the primary concern. The magnICON system also illustrated the importance of agroinfiltration for the delivery of a deconstructed vector, as an earlier attempt to introduce a TMV vector lacking the viral CP through the use of RNA transcripts (Takamatsu *et al*., 1987) gave only very low levels of foreign gene expression. In the magnICON system, the 3′ module contains the gene of interest in place of the sequence of the viral CP along with the *nos* terminator, the 5′ module contains the *Arabidopsis* actin 2 (ACT2) promoter along with the tobamovirus polymerase and movement protein genes (the latter of which contains the native CP subgenomic promoter), while the recombinase module containing the PhiC31 integrase gene (from *Streptomyces* phage C31) acts to fuse the 3′ and 5′ modules together in the nucleus to allow the formation of a complete tobamovirus‐based replicon, with the GOI under the control of the subgenomic promoter present within the MP sequence. The RNA is then able to replicate, spread from cell to cell within the leaf (though not throughout the entire plant as the CP gene has been deleted) and produce high levels of the protein of interest. In proof‐of‐concept experiments, yields of GFP as high as 5 mg/g of fresh weight tissue (5 mg/g FWT) are reported (Marillonnet *et al*., 2004). A subsequent optimization of the tobamovirus sequences, including the addition of numerous introns, vastly improved the efficiency with which active replicons are formed from agroinfected cells, thus reducing the reliance on agroinfection efficiency, so a successful infection can be initiated from a very dilute suspension of *Agrobacterium* (OD_600_ as low as 0.0035). These updated vectors were shown to be capable of yielding up to 4 mg/g FWT of GFP (Marillonnet *et al*., 2005).

The modular nature of the magnICON system enabled a variety of different 3′ modules incorporating different targeting sequences to be used to direct the expressed protein to a variety of cellular compartments (Marillonnet *et al*., 2004). In this case, a single 3′ module containing the GOI can be co‐infiltrated with many different 5′ modules (on different leaves) which each contain a different targeting signal. However, when Gils *et al*. (2005) used the magnICON system to produce human growth hormone in plants and target the protein to either the cytosol, apoplast or chloroplast, they used three different 3′ modules containing three slightly different sequences, and a single 5′ module. In any case, any advantage of such a three‐module system (which also requires the co‐infiltration of three different agrobacterial suspensions) has been lessened in recent years by the advent of rapid gene synthesis permitting the facile incorporation of targeting sequences and/or cloning sites into any synthetic gene. Given this, it is difficult to see the advantage of the triple‐module system over a single construct for efficient infection of plants. Indeed, the original description of the magnICON system included a description of a pre‐assembled (single module) vector that seemed to yield slightly more recombinant protein than the triple‐module version (Marillonnet *et al*., 2004), and the authors suggested that ‘pre‐assembled complete viral vectors’ could be an improvement to the magnICON system for scale‐up of recombinant protein production. However, such single‐module magnICON vectors only seem to have been used twice (Hamorsky *et al*., 2013; Marillonnet *et al*., 2005).

The triple‐module magnICON system has been used to produce a wide variety of proteins in plants, including plague antigens which reached yields of 2 mg/g FWT (Santi *et al*., 2006), hepatitis B virus core antigen (HBcAg) virus‐like particles (VLPs) at up to 2.4 mg/g FWT (Huang *et al*., 2006) and Norwalk virus VLPs at 0.8 mg/g FWT (Santi *et al*., 2008). The production of antibodies was, for a time, more problematic. Soon after the development of the magnICON system, it became clear that viral competition would make it difficult to co‐express multiple replicons in the same cells, which would make it impossible to produce oligomultimeric proteins like immunoglobulin G (IgG) (Gleba *et al*., 2005). The solution to this problem was to express one chain of the IgG using magnICON, and the other chain using a different noncompeting viral vector system, based on potato virus X (PVX). This allowed the production of assembled IgG in *N. benthamiana* with yields reaching 0.5 mg/g FWT (Giritch *et al*., 2006). Since then, IgG have been produced using this noncompeting viral vectors strategy in an industrial setting as candidate vaccines for hon‐Hodgkin's lymphoma (Bendandi *et al*., 2010). An alternative strategy, developed by Roy *et al*. (2010), involves using a defective RNA‐based TMV vector (dRT‐V). This system is composed of two components: a helper TMV‐based deconstructed construct carries the GOI in the place of the CP, while the second GOI is placed on a second TMV‐based construct in which a large deletion in the replicase and movement protein makes the two constructs noncompetitive: the helper construct provides the replication and movement proteins required for itself and the dRNA, thus allowing co‐expression of two GOIs in the same cells. Using this system, a monoclonal IgG specific to the protective antigen of *Bacillus anthracis* was produced in *N. benthamiana*, with yield reaching 0.12 mg/g FWT. However, it was noted that there was a significant difference in the respective accumulation levels of the heavy (H) and light (L) chains.

Another obvious way of solving the competition issue is to co‐express the different polypeptide chains in *cis* from the same T‐DNA. This was attempted by Roy *et al*. (2011), who successfully co‐expressed two GOIs from a single deconstructed TMV vector using two different subgenomic promoters: the first GOI is under the control of the native TMV U1 CP subgenomic promoter, while the second is under the control of the heterologous CP subgenomic promoter from the related tobacco mild green mosaic virus U5. However, accumulation of co‐expressed recombinant protein was relatively modest (0.05–0.2 mg/g of GFP and DsRed). A bicistronic approach was reported by Hamorsky *et al*. (2013) to produce an anti‐HIV IgG (VRC01) in *N. benthamiana*. In this study, the sequences for the heavy (H) and light (L) chains were fused together via a linker containing an endoprotease cleavage site on a single TMV‐based deconstructed vector. Upon expression of the 2‐kb sequence and trafficking of the propeptide through the endoplasmic reticulum and Golgi, the propeptide was processed and the H and L chains assembled to form functional IgG. The reported yield of purified IgG was 0.15 mg/g FWT. This is lower than the yield obtained by Giritch *et al*. (2006), but similar to the yield obtained by Roy *et al*. (2010) with noncompeting viral vector systems. However as these studies aimed to produce different IgG, it is difficult to draw any conclusions on the relative merits of each approach. There may also be limits on the size of the GOI beyond which the replication rate of the RNA or its genetic instability becomes a problem. These issues are not restricted to TMV‐based systems but are an inherent disadvantage of all replicating RNA virus‐based vector systems.

Subsequent to the first reports on magnICON, a single‐module TMV‐based overexpression vector was developed. The TRBO system (Figure 1b) is based on a 35S promoter‐led replicon based on the TMV genome from which the CP gene (under the control of the native subgenomic promoter present within the MP gene) is replaced with a multiple cloning site into which the GOI is inserted (Lindbo, 2007). Like magnICON, TRBO replicons cannot move systemically, but they can move cell to cell within an infected leaf, so agroinfiltration is crucial to initiating an infection of the entire plant. This simple design was tested with GFP as the GOI, and it was produced at up to 5.5 mg/g FWT. Interestingly, the author demonstrated that the co‐expression of an exogenous suppressor of gene silencing had no effect on transgene expression. However, this was not the case for versions of the expression vector that still contained the CP gene, which led the author to suggest that this sequence may be an inducer of gene silencing. Moreover, it has since been determined that suppressor of silencing activity is in fact already present on the replicase protein of TMV (Wang *et al*., 2012). In addition to this, the initiation of primary replicons in agroinfected cells seemed to be just as efficient as the improved magnICON vectors, but without the need to insert numerous exogenous sequences such as introns into the tobamovirus genes. The author suggests that this may be due to the fact that TRBO is based on the U1 strain of TMV, while magnICON is based on TVCV and the crucifer‐infecting strain of TMV (cr‐TMV). It therefore appears that a TRBO vector has all of the advantages of a magnICON vector, but is smaller and simpler in design. However, there are no published reports of side‐by‐side comparisons of magnICON and TRBO. Along with GFP, Lindbo (2007) demonstrated the usefulness of the TRBO system with *Phytophthora infestans* Avr3a, *Arabidopsis* adenosine kinase, the FN10 domain from human fibronectin and tomato RCR‐3 and P69b proteinases. While these are all overexpressed, they do not accumulate to titres as high as GFP, with the notable exception being *Arabidopsis* adenosine kinase. A chimaeric antigen derived from house dust mites was also produced using TRBO, and this accumulated to up to 4.5 mg/g FWT (Li *et al*., 2013). Together, these data suggest that the TRBO system is comparable to magnICON in terms of recombinant protein yield and may be simpler to use.

## Potexvirus‐based vectors

The development of potexvirus‐based vectors is very similar to that of tobamovirus‐based vectors described above. Potexviruses, of which potato virus X (PVX) is the type species, are flexuous rod‐shaped +ssRNA viruses. An early example of the use of PVX as an expression system involved the insertion of the GFP gene upstream of the CP gene either as a separate ORF or as a fusion protein linked by a self‐cleaving 2A peptide from foot‐and‐mouth disease virus (FMDV) (Baulcombe *et al*., 1995; Santa Cruz *et al*., 1996). Both strategies worked, producing either free GFP or chimaeric PVX rods displaying GFP on the surface linked to some of the CPs, thus demonstrating the potential for PVX to be used as an expression system and as a display scaffold for whole proteins of interest. This second application was further explored by Smolenska *et al*. (1998), and O'Brien *et al*. (2000), who used the 2A peptide fusion strategy to display (respectively) a functional single‐chain variable fragment antibody, and the 45‐kDa VP6 protein of rotavirus, on the surface of PVX. Without using the 2A peptide, Marusic *et al*. (2001) displayed a six amino acid‐long epitope of human immunodeficiency virus (HIV) at the N‐terminus of PVX CP, which yielded chimaeric virus which displayed the epitope on their surface. Further research defined the limits as to which peptides could be presented in this manner (Lico *et al*., 2006).

**Figure 2:**
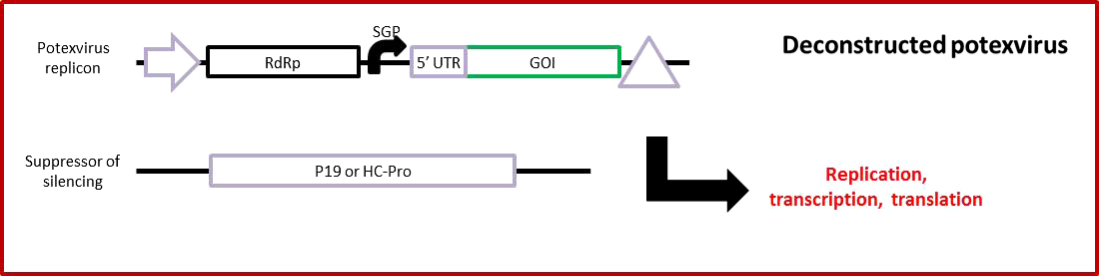
Simplified diagram of a fully deconstructed potexvirus‐based expression system. The potexvirus coat protein and movement protein genes are replaced with the gene of interest (GOI) under the control of a native viral triple gene block (TGB) subgenomic promoter (SGP) downstream of the viral RNA‐dependent RNA polymerase (RdRp). The replicon can be placed under the control of the native viral promoter and terminator (Komarova *et al*., 2006), or the 35S promoter and CaMV terminator (Liu and Kearney, 2010). A translational enhancer can be added upstream of the GOI, such as the 5′ UTR of RNA‐4 of alfalfa mosaic virus (Mardanova *et al*., 2009), or the first 40 nucleotides of the potexvirus TGB open reading frame with a mutated start codon (Liu and Kearney, 2010). Expression is enhanced by co‐infiltrating a suppressor of silencing (P19 from TBSV or HC‐Pro from PVA). Such replicons are capable of replication, transcription and translation, but not cell‐to‐cell movement.

At around the same time as tobamovirus genomes were being deconstructed, similar work was being done on PVX. Komarova *et al*. (2006) replaced the CP gene and triple gene block (TGB) with GFP, which was under the control of the native TGB subgenomic promoter SGP1 (Figure 2). Because both the CP and the three TGB proteins are required for viral movement (Batten *et al*., 2003; Beck *et al*., 1991), there was indeed no obvious reason to retain the TGB once the CP had been removed. The result is a replicating vector that is significantly smaller than the original PVX genome and incapable of cell‐to‐cell movement, a feature which is compensated for by the efficiency of agroinfiltration. This vector clearly yielded large quantities of GFP, especially when the potato virus A (PVA) suppressor of gene silencing (HC‐Pro) was supplied along with the PVX replicon. This system was improved when the 5′ untranslated region (UTR) of RNA‐4 of alfalfa mosaic virus (AMV) was added between SGP1 and the GFP ORF (Mardanova *et al*., 2009). This translational enhancer increased GFP accumulation three to fourfold, demonstrating the usefulness of translational enhancers, the deployment of which is often overlooked when designing expression vectors. Another improvement was provided when PVX strain Tula was used as the basis of the deconstructed vector instead of the previously used strain UK3 (Ravin *et al*., 2008). A side‐by‐side comparison indicated that the Tula‐based vector yielded 1.5–2 times higher GUS expression than the UK3‐based vector. This demonstrates, as did the comparison of TRBO and magnICON, that some strains of virus are better suited for the development of expression vectors than others. While it is clear that these PVX‐based vectors yield large amounts of recombinant proteins, the lack of absolute quantification in the articles cited makes it difficult to compare them to other expression systems. A similarly deconstructed PVX vector was developed for the expression of recombinant proteins in cell suspension cultures (Larsen and Curtis, 2012), and while this did lead to transgene expression, the authors concluded that such a system was far better suited to expression in leaves.

The fact that deconstructing the PVX genome greatly reduces its size leaves open the possibility of co‐expressing multiple GOI from the same PVX replicon. In theory, this can be done relatively easily by adding subgenomic promoters to the replicon upstream of each new GOI. In practice, there do not appear to be any published examples of this. Wang *et al*. (2014) did create a PVX‐based vector called pCaPVX440 in which GFP and YFP are co‐expressed from two different subgenomic promoters, but these are located between the TGB and CP, which are both retained in the vector. This vector allowed co‐expression of the two fluorescent proteins, but the expression levels seemed to be quite low. A version of the vector (called pCaPVX760) in which the CP was deleted allowed for higher yield of recombinant GFP, but co‐expression of two proteins was not tested with this vector. Moreover, pCaPVX760 retains the TGB even though movement is abolished due to the absence of CP, which indicates that there may be room for improvement of this vector system. While removing both the CP and the TGB would quite possibly improve yield of co‐expressed proteins, there may be genetic instability issues associated with the addition of extra genes and subgenomic promoters. One would still expect this to be an issue even with fully deconstructed PVX‐based vectors, because genetic instability in PVX is mainly associated with replication, and not encapsidation as is often the case with icosahedral viruses that have limited space inside the viral capsid. To address this concern, Dickmeis *et al*. (2014) developed a nondeconstructed PVX‐based vector which contains an added subgenomic promoter from bamboo mosaic virus downstream of the TGB, and an N‐terminally truncated CP. This vector is genetically stable over multiple passages without abolishing movement or encapsidation of the modified RNA.

It seems that co‐expression of multiple GOIs from a single fully deconstructed PVX‐based vector has never been attempted. Given the work described above, it seems likely that two GOIs could be co‐expressed from a PVX vector in which the CP and TGB have been removed, thanks to the efficiency of agroinfiltration which eliminates the need for cell‐to‐cell movement. If genetic instability proves to be an issue with such a vector, heterologous subgenomic promoters such as those described in Dickmeis *et al*. (2014) could be employed to stabilize the replicon. Such a system could simplify the production of oligomeric proteins from replicating vectors and may even prove to be an improvement on the TMV/PVX noncompeting vector system described above.

As discussed above, the choice of virus strain can make a significant difference in the quality of the expression system based on that strain. Similarly, the choice of viral species within a taxon can also make a difference. While the expression systems described above are based on PVX, Sempere *et al*. (2011) created a viral vector based on pepino mosaic virus (PepMV), a different potexvirus. The authors tested different strategies to determine which gave the highest yield of GFP. They found that the optimal use of the vector involved fusing GFP to the N‐terminus of the CP, which resulted in GFP yields of 0.2–0.4 mg/g FWT, which is inferior to most other replicating viral overexpression systems. Similarly, Zhang *et al*. (2013) created a nondeconstructed expression vector based on the potexvirus narcissus mosaic virus (NMV), which allowed for systemic spread of transgenic viral RNA, but only succeeded in initiating low numbers of small infection foci in which transgene expression was relatively low. On the other hand, Liu and Kearney (2010) developed the FECT vector series based on fully deconstructed foxtail mosaic virus (FoMV), yet another potexvirus (Figure 2). This system is analogous to that described above (Komarova *et al*., 2006): in the highest‐yielding FECT/40 vector, the CP and TGB are deleted, and the GOI is placed under the control of the native TGB subgenomic promoter, with the first 40 bp of the TGB1 ORF (with ATG start codon mutated to ATC) acting as a 5′ translational enhancer. In a side‐by‐side comparison with TRBO (Lindbo, 2007), FECT/40 was just as efficient at producing GFP, with yields as high as 1.7 mg/g FWT. This is particularly impressive given that FECT/40 cannot move cell to cell, but TRBO can, which demonstrates the extent to which agroinfiltration efficiency compensates for deficiencies in viral movement. Interestingly, such high yield with FECT/40 is entirely dependent on the co‐expression (in *cis* on the same T‐DNA or in *trans* on a different T‐DNA) of the P19 suppressor of silencing from tomato bushy stunt virus (TBSV). Without P19, expression of GFP in leaves was found to be almost unnoticeable to the naked eye (Figure 4 in Liu and Kearney, 2010). The authors point out that this characteristic could be advantageous for creating an inducible expression system either in a stable transgenic context or for the production of toxic proteins. Moreover, the authors make use of the wide host range of FoMV to test the FECT expression system in various monocotyledonous plants, including barley, wheat and maize. In these cases, however, agroinfiltration efficiency was very low, leading to very low transgene expression. The remarkable difference in the efficiency of the FECT vector in dicots as compared to monocots is indicative of the crucial importance that agroinfiltration has in the success of a deconstructed viral vector. The development of expression systems for monocots is discussed in more detail below.

## Tobravirus‐based vectors

**Figure 3:**
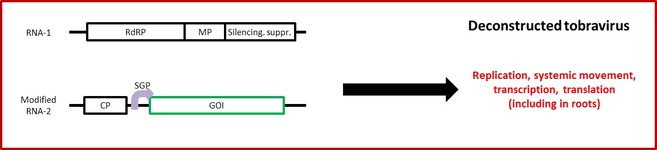
Simplified diagram of a deconstructed tobravirus‐based expression system. The RNA‐1 component supplies the viral RNA‐dependent RNA polymerase (RdRp), movement protein (MP) and native suppressor of silencing, while the modified RNA‐2 contains the viral coat protein gene (CP), but the genes required for nematode transmission are replaced with the gene of interest (GOI) under the control of a heterologous CP subgenomic promoter (SGP) from a different tobravirus (MacFarlane and Popovich, 2000). Such replicons are capable of replication, cell‐to‐cell and systemic movement, transcription and translation in both leaves and roots.

Tobraviruses are rod‐shaped bipartite +ssRNA viruses. The RNA‐1 component carries the viral replicase, movement protein genes and suppressor of silencing, whereas RNA‐2 codes for the CP and genes necessary for nematode transmission. The latter genes are not required for viral infection *per se*, so numerous groups have developed tobravirus‐based vectors in which the GOI replaces these nematode transmission genes, a strategy which has been particularly successful for the development of virus‐induced gene silencing (VIGS) vectors (Constantin *et al*., 2004; Deng *et al*., 2013; MacFarlane, 2010). For protein overexpression, tobravirus‐based vectors have proved to be capable of stable systemic infection in *N. benthamiana*. This allowed Yang *et al*. (2013) to transiently create cold‐tolerant *N. benthamiana* by transiently expressing calmodulin from an Antarctic notothenioid fish. Importantly, it was also demonstrated that a tobacco rattle virus (TRV)‐based vector could direct the expression of GFP not just in leaf tissue, but also in the root system of *N. benthamiana*, with far greater efficiency than a PVX‐based vector (MacFarlane and Popovich, 2000; Figure 3). The authors then showed that the same vector could be used to express the *Galanthus nivalis* agglutinin GNA lectin protein in *N. benthamiana* roots, with yield reaching 10 μg/g of fresh weight root tissue. Importantly, expression of the protein was stable in roots for at least 24 days (with peak expression at 6–9 days), and over at least four serial passages.

In all of the studies cited above, tobravirus‐based vectors were designed so that the nematode transmission genes were removed, but the coat protein gene was always retained. It is not immediately clear why this was the case. It meant that MacFarlane and Popovich (2000) had to use CP subgenomic promoters from other tobraviruses to direct the expression of their transgenes in order to avoid genetic instability due to sequence duplication. Moreover, it is known that the tobravirus CP is dispensable for viral replication and movement (MacFarlane, 2010; Swanson *et al*., 2002). However, it could be that retaining the CP favours co‐infection of the wild‐type RNA‐1 with the modified RNA‐2. Indeed, there is some suggestion that in the absence of the CP, RNA‐1 and RNA‐2 separate, resulting in a ‘nonmultiplying’ infection consisting solely of RNA‐1 (Swanson *et al*., 2002). Because RNA‐2 contains the transgene, it may be necessary, or at least useful, to maintain the CP to avoid the loss of RNA‐2 during systemic spread.

## Geminivirus‐based vectors

Unlike the viruses described above, geminiviruses are twin‐spherical viruses with single‐stranded circular DNA genomes. This presents certain advantages over RNA viruses: firstly, geminivirus genomes were easier to manipulate before modern molecular biology techniques such as RT‐PCR and *in vitro* transcription became standard, and secondly, the circular DNA genome can handle far larger insertions than linear RNA genomes. Thus, much of the early work on developing virus vectors focussed on creating episomally replicating geminiviral constructs (reviewed by Porta and Lomonossoff, 2002).

The long and short intergenic regions (LIR and SIR, respectively) on the geminivirus genome allow a linear construct to be circularized by joining of two LIR sequences by the viral Rep/RepA proteins. Mor *et al*. (2003) demonstrated that this allows the deployment of ‘LSL’ (LIR SIR LIR) vectors, in which a linear construct based on a deconstructed bean yellow dwarf virus (BeYDV) containing a SIR and flanked by two LIR can be delivered into plants, and will then circularize to form replicons. The circularization and replication is dependent upon the action of the Rep protein, which can be supplied in *trans*. This allows separate regulation of the GOI (on the LSL replicon) and Rep, which allows for greater control of the potential toxicity that is sometimes seen with Rep. The authors suggested that this system might be adapted to create lines of stably transformed plants, in which the GOI‐containing LSL construct and an inducible Rep cassette are stably transformed into plants, and the GOI is expressed from circular replicons upon induction of Rep. This suggestion was taken up by Zhang and Mason (2006) who developed an LSL expression system for tobacco cell cultures and stable transgenic potato. In this system, Rep is under the control of the *Aspergillus nidulans* ethanol‐inducible promoter. The GOI is expressed at low levels constitutively, but upon ethanol induction, yields of Norwalk virus CP (NVCP) increased 2‐ to 10‐fold in tobacco cell culture, and yields of GFP increased eightfold in transgenic potato. The authors indicated that this system is less prone to silencing and toxicity than other similar geminiviral systems in which Rep is constitutively expressed. However, transgene expression decreases in transgenic potato 8 days after induction, suggesting that silencing does eventually take place. The authors suggest that the system might be improved by the addition of a suppressor of gene silencing, as well as by the addition of translational enhancers: indeed, upon ethanol induction, mRNA levels increase far more than protein expression, which suggests that there is room for optimization of translational efficiency.

**Figure 4:**
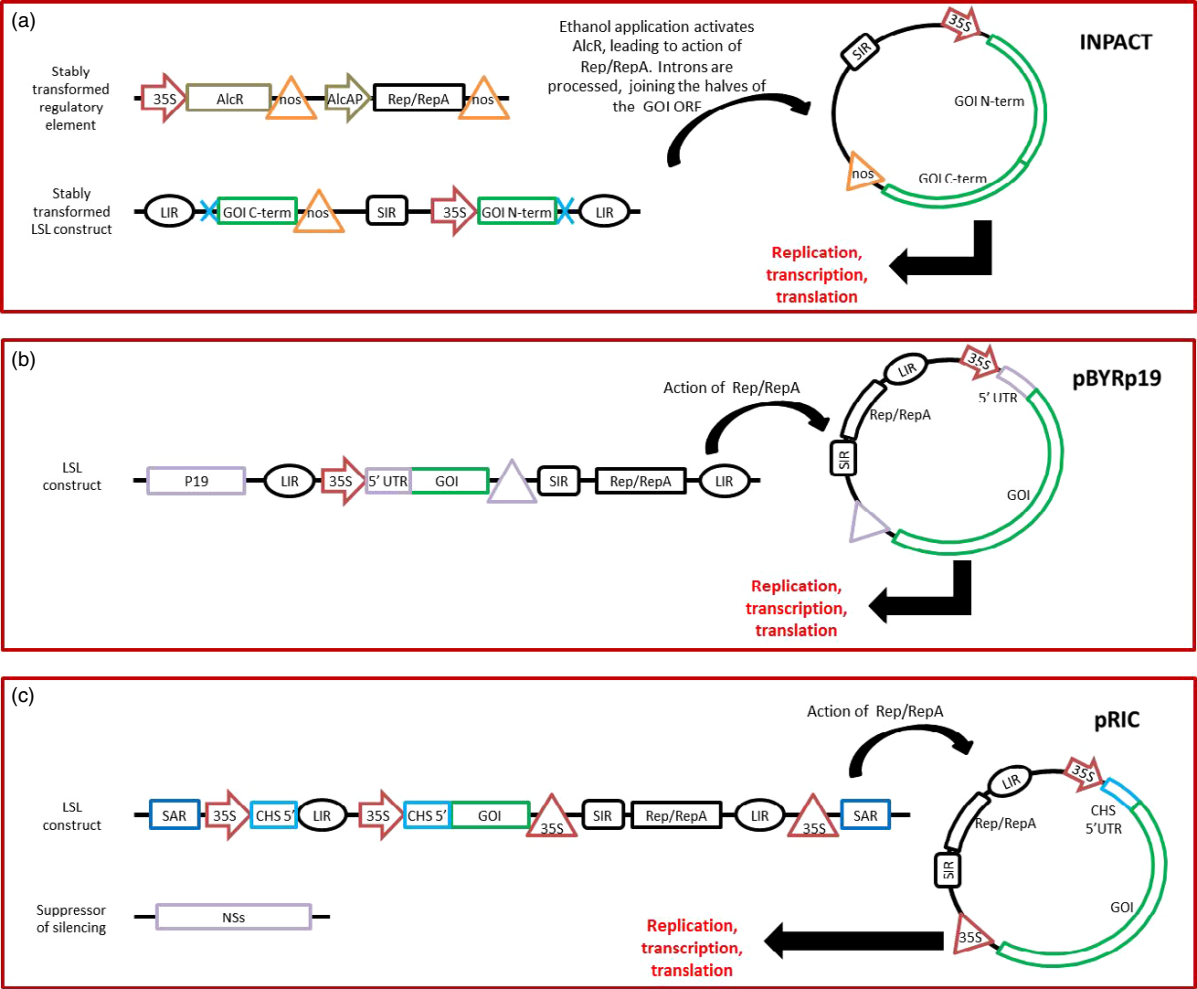
Simplified diagrams of deconstructed geminivirus‐based expression systems. (a) The INPACT system (Dugdale *et al*., 2013) is composed of two elements which are transformed stably into the plant genome. The regulatory element contains ethanol‐inducible Rep/RepA gene, while the LSL construct contains the gene of interest (GOI) split in half with introns immediately upstream of the C‐terminal half and immediately downstream of the N‐terminal half. When activated by ethanol, Rep/RepA circularize the LSL construct at the LIRs, and processing of the introns leads to loss of the LIR and recombination of the two halves of the GOI open reading frame. 35S arrow: 35S promoter, AlcR: ethanol‐activated transcription factor, nos triangle: nos terminator, AlcAP arrow: AlcR‐activated promoter, LIR: long intergenic region, blue cross: introns, SIR: short intergenic region. (b) The pBYRp19 system (Chen *et al*., 2011) is composed of a single construct carrying the TBSV P19 silencing suppressor as well as an LSL which contains both the Rep/RepA functions and the GOI. There are multiple versions of the vector: in pBYR1, the translational enhancer (5′ UTR) is the tobacco etch virus 5′ UTR and the terminator (triangle) is soya bean vspB 3′ region, whereas in pBYR2, the translational enhancer is TMV Ω, and the terminator is the extensin gene 3′ region. (c) The pRIC system (Regnard *et al*., 2010) is composed of an LSL replicon inserted into the multiple cloning site of a pTRAc vector. The pTRAc vector T‐DNA is flanked by SAR elements (scaffold attachment region of the tobacco Rb7 gene), and the LSL contains the Rep/RepA functions as well as the GOI, which is under the control of the 35S promoter (arrow) and terminator (arrow), with chalcone synthase 5′ UTR as a translational enhancer (CHS 5′). The NSs silencing suppressor from tomato spotted wilt virus is typically co‐infiltrated. Upon action of Rep/RepA, all of these expression systems form circular replicons that are capable of replication, transcription and translation, but not cell‐to‐cell or systemic movement.

A similar approach was used to develop the in‐plant activation (INPACT) expression system (Dugdale *et al*., 2013), which is based on stably integrated components of tobacco yellow dwarf mastrevirus (TYDV, Figure 4a). The Rep/RepA cassette is under the control of an ethanol‐inducible promoter, and the GOI is split on either side of the SIR, while the LIR are flanked by introns. Upon ethanol induction, Rep/RepA circularizes the LSL, which forms a circular replicon. The GOI is transcribed from this replicon, and processing of the intron joins the two halves of the GOI ORF, allowing translation of the ORF. This allows very tight control of the GOI, which cannot be expressed until ethanol induction. However, peak transgene expression takes place at 3–4 days postethanol induction, which strongly suggests that gene silencing is limiting the potential of this system. As with the system described by Zhang and Mason (2006), the system could probably be improved by the addition of a suppressor of silencing and a translational enhancer.

The Mason group continued to improve BeYDV‐based vectors for transient expression and developed a new system which was intended to be more practical than previous versions. The stated goal was to develop a system which would provide transgene expression at levels comparable to magnICON, but without the need to co‐infiltrate at least three different agrobacterial suspensions (Huang *et al*., 2009). In this new system, the GOI is placed between a tobacco etch virus (TEV) 5′ UTR and soya bean vspB 3′ region for improved translational efficiency within the LSL. The authors then tested the optimal way of providing Rep/RepA. They determined that highest yield of transgene (GFP, HBcAg or NVCP) was obtained when Rep/RepA was provided in *cis* within the LSL under the control of the relatively weak promoter activity of the LIR, as opposed to in *trans* on a different nonreplicating T‐DNA under the control of the stronger 35S promoter. Co‐infiltration of a construct carrying the TBSV P19 suppressor of silencing further increased yield. Using the configuration with which Rep/RepA is provided in *trans*, HBcAg was expressed up to 0.8 mg/g FWT and NVCP was expressed up to 0.34 mg/g FWT.

This system was then shown to be capable of co‐expressing two GOIs from a double LSL construct which share the same T‐DNA (Huang *et al*., 2010): upon action of Rep/RepA, the double LSL forms two separate circular replicons, each of which overexpresses one GOI. This was successfully deployed to produce 0.5 mg/g of the anti‐ebola monoclonal IgG 6D8 in *N. benthamiana*, which is comparable to expression levels of a different IgG obtained by Giritch *et al*. (2006), who had to combine the three‐component magnICON system with a PVX‐based vector. In their publication, Huang *et al*. (2010) suggested that their BeYDV expression system may be further improved by adding P19 in *cis* (they had so far been supplying it in *trans*), and by replacing the TEV 5′ leader with the ‘hypertranslatable’ cowpea mosaic virus (CPMV)‐based CPMV‐*HT* 5′ leader (Sainsbury and Lomonossoff, 2008; see below). Chen *et al*. (2011) carried out the former modification to develop user‐friendly BeYDV‐based vectors (Figure 4b). The pBYR1 and pBYR2 vectors are each composed of a T‐DNA containing a kanamycin resistance cassette and an LSL that includes a multiple cloning site (MCS) flanked by one of two different sets of UTRs, along with Rep/RepA under the control of LIR as a promoter. The pBYR1p19 and pBYR2p19 vectors are identical to pBYR1 and pBYR2 except that the kanamycin resistance cassette is replaced by a P19 cassette.

Another similar user‐friendly vector system is the BeYDV‐based pRIC expression system (Regnard *et al*., 2010; Figure 4c). This is essentially a replicating version of the pTRAC system (Maclean *et al*., 2007), which makes use of the scaffold attachment region of the tobacco Rb7 gene (SAR) to enhance transgene expression (Halweg *et al*., 2005). This pRIC expression system allowed overexpression of human papillomavirus (HPV) CP L1 (0.55 mg/g FWT) and HIV capsid protein p24 (3.23 μg/g FWT). The translational efficiency of this system could probably be improved, given that replication enhanced transgene copy number up to 1000‐fold, but protein expression only increases by up to sevenfold.

BeYDV replicons have also been proposed as efficient vehicles of targeted genome engineering thanks to the transient expression of LSL replicons containing zinc finger nucleases or CRISPR/CAS elements for highly efficient targeting of transgenes or mutagenesis (Baltes *et al*., 2014). Another geminivirus, beet curly top virus (BCTV), has been used to develop a replicating expression system (Kim *et al*., 2007). In this system, the GOI is placed under the control of the cassava vein mosaic virus (CsVMV) promoter, which was found to lead to three times higher transgene expression than the 35S promoter. This system was then used to produce hepatitis A virus VP1 protein which was fused to an IgG constant fragment (Fc) region, but expression levels were relatively modest (Chung *et al*., 2011).

## Comovirus‐based vectors

Comoviruses are a genus of bipartite positive‐strand RNA viruses. Most of the work on vector development has concerned the type member, cowpea mosaic virus (CPMV: Sainsbury *et al*., 2010a), although vectors based on bean pod mottle virus (BPMV) have also been developed (Zhang *et al*., 2010). In every case, vector construction has involved modifications made to the smaller genomic RNA (RNA‐2) which encodes the structural and movement proteins. When required to provide replication functions, unmodified RNA‐1 is supplied.

As with most RNA virus‐based vector systems, the early development of CPMV‐based vectors involved adding additional sequences to full‐length RNA‐2 and then mechanically inoculating plants with either *in vitro* transcripts or 35S‐driven constructs in the presence of RNA‐1. This method proved successful both for the production of assembled CPMV particles displaying epitopes on their surface (Porta *et al*., 1994; Usha *et al*., 1993) and for the production of polypeptides (Gopinath *et al*., 2000). For epitope display, this mechanical infection approach was successful in producing chimaeric CPMV particles containing epitopes from a variety of animal pathogens, and particles presenting a peptide from mink enteritis virus (MEV) were shown to be capable of protecting mink against challenge with MEV (Dalsgaard *et al*., 1997). This represented the first example of a candidate vaccine produced using a plant virus‐based vector. In the case of recombinant proteins, these could be released from the RNA‐2‐encoded polypeptide through the action of either the virus‐encoded 24K protease or a FMDV 2A catalytic peptide (Gopinath *et al*., 2000). The 2A‐mediated approach has been used to express HBcAg (Mechtcheriakova *et al*., 2006) small immune proteins (Monger *et al*., 2006) and the H and L chains from the blood‐typing antibody, C5‐1 (Sainsbury *et al*., 2008).

As with other vectors based on full‐length replicating RNA molecules, the approach adopted above suffered from several disadvantages. The use of transcripts or DNA to initiate an infection meant that it was essential that the virus retained its ability to spread if reasonable yields were to be obtained. This became an issue as it was found that the expression of long or basic peptides on the viral surface compromised this ability resulting in poor or negligible yields of particles being obtained (Porta *et al*., 2003). Likewise, it was found that sequences encoding polypeptides tended to be deleted during virus spread (Gopinath *et al*., 2000), and the segregation of RNA‐2 constructs expressing different proteins was found to occur during systemic spread (Sainsbury *et al*., 2008).

**Figure 5:**
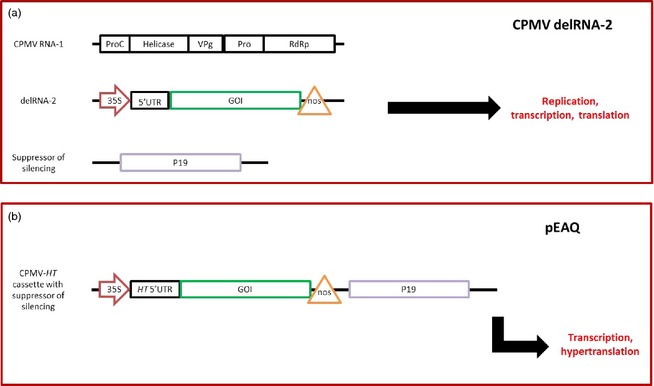
Simplified diagrams of comovirus‐based expression system. (a) In the delRNA‐2 system (Cañizares *et al*., 2006), the wt CPMV RNA‐1, which contains the replication machinery, is co‐infiltrated with the modified RNA‐2 (delRNA) along with a suppressor of silencing (P19 from TBSV). ProC: proteinase cofactor, VPg: genome‐linked protein, Pro: proteinase, RdRp: RNA‐dependent RNA polymerase, 35S arrow: 35S promoter, 5′ UTR: CPMV RNA‐2 5′ untranslated region, GOI: gene of interest, nos triangle: nos terminator. The delRNA‐2 system is capable of replication, transcription and translation, but not cell‐to‐cell or systemic movement. (b) The pEAQ series of vectors contain T‐DNA constructs that contain a CPMV‐HT cassette, in which the GOI is placed downstream of the hypertranslatable CPMV‐HT 5′ UTR. The CPMV‐HT construct is not capable of replication or movement and instead relies on transcription and extremely efficient translation.

The advent of an agroinfection system for CPMV (Liu and Lomonossoff, 2002) enabled the possibility of developing CPMV vectors based on deconstructed versions of RNA‐2 to be explored (Cañizares *et al*., 2006). It was found that replacement of the entire open reading frame of RNA‐2 with GFP still permitted replication of the modified RNA‐2 by RNA‐1, provided that the sequence of 512 nucleotides from the 5′ end of RNA‐2 and the entire 3′ UTR were retained (delRNA‐2, Figure 5a). It was also necessary to co‐infiltrate a suppressor of gene silencing, such as HcPro or P19, as deletion of the RNA‐2 open reading frame removed the natural CPMV suppressor of gene silencing, the small CP (Liu *et al*., 2004). The replication‐competent deleted versions of RNA‐2 proved successful as vectors, allowing the expression of the C5‐1 antibody and HBcAg particles (Meshcheriakova *et al*., 2008; Sainsbury *et al*., 2008). However, during these studies it was noticed that the levels of expression from the deleted RNA‐2 constructs in the presence of the strong silencing suppressor P19 (from TBSV) differed little in the presence or absence of RNA‐1, presumably as a result of the mRNA being so highly stabilized in the presence of the suppressor that replication was unnecessary to maintain high mRNA levels. Thus for several applications, such as the expression of metabolic enzymes in *N. benthamiana*, RNA‐1 was not co‐infiltrated (Mugford *et al*., 2009, 2013).

The observation that replication was not essential for expression from the deleted RNA‐2 constructs led Sainsbury and Lomonossoff (2008) to study whether the structure of the sequence 5′ to the main initiation codon at position 512 could be simplified as an aid to cloning. Removing two upstream initiation (AUG) codons at positions 115 and 161 had the unexpected effect of enhancing protein expression levels 10‐ to 15‐fold compared to those obtainable with the original deleted RNA‐2 construct containing the wild‐type 5′ sequence. This effect was shown to be due to enhanced levels of mRNA translation, and so constructs containing the modified RNA‐2 5′ sequence were termed CPMV‐*hypertanslatable* or CPMV‐*HT*; it was subsequently shown that the 3′ UTR also plays an important role in ensuring high levels from CPMV‐*HT* constructs (Meshcheriakova *et al*., 2014). A series of user‐friendly vectors, the pEAQ series, allowing the facile insertion of sequences between the 5′ and 3′ sequences of a CPMV‐*HT* cassette were subsequently developed (Sainsbury *et al*., 2009). These also had a modular design permitting several CPMV‐*HT* cassettes and the TBSV P19 suppressor of silencing to be expressed from a single T‐DNA (Figure 5b).

Obviating the need for replication gives the CPMV‐*HT* system and its associated pEAQ vectors a number of advantages over systems based on replicating RNA virus‐based vectors: there appears be no limit of the size of gene that can be expressed, there is no issue of virus exclusion in the absence of replication, and the expression levels of individual proteins can be modulated. As a result, the pEAQ vectors have been used to express a variety of proteins in plants (Peyret and Lomonossoff, 2013; Sainsbury and Lomonossoff, 2014). In terms of proteins of interest to molecular farming, although some work has been carried out on antibody (Sainsbury *et al*., 2010b) and therapeutic enzyme expression (Vardakou *et al*., 2012), much of the research using the pEAQ vectors has focussed on the production of VLPs because of their potential as candidate vaccines (Thuenemann *et al*., 2013b). This work has included the production of VLPs consisting of only a single polypeptide, such as the papillomaviruses (Love *et al*., 2012; Matić *et al*., 2012), or more complex structures. An example of the latter is bluetongue virus (BTV), VLPs of which consist of four separate polypeptides present at different stoichiometries. The nonreplicative nature of the pEAQ‐*HT* vector meant that it was possible to express each of the four proteins at the required level within the same cell, thereby maximizing the level of correctly assembled VLPs (Thuenemann *et al*., 2013a); the purified VLPs proved to be capable of stimulating a protective immune response in sheep. It has also proved possible to produce VLPs from enveloped viruses using the CPMV‐*HT* system, most notably influenza virus (D'Aoust *et al*., 2008); plant‐produced VLPs consisting of membrane vesicles expressing the viral haemagglutinin have successfully completed phase II clinical trials (Landry *et al*., 2010).

In a clear demonstration of the convergence of *Agrobacterium*‐based transformation methods and virus‐based vectors, the pEAQ plasmids have been used to create stably transformed lines of either whole *N. benthamiana* plants (Saxena *et al*., 2011) or BY‐2 tobacco cells in culture (Sun *et al*., 2011). While an unmodified version of the pEAQ system could be used to transform BY‐2 cells with a construct expressing human serum albumin (HSA), the regeneration of fertile transgenic *N. benthamiana* required the P19 sequence within the vector to be modified to reduce its silencing suppression activity.

## A word about monocots

The vast majority of efforts (and successes) in the transient overexpression of proteins of interest in plants have been achieved with dicotyledonous plants. However, that is not to say that monocotyledonous plants have been completely ignored. Cell cultures of graminaceous plants have been used as hosts for the expression of one or more transgenes expressed from geminivirus vectors based on wheat dwarf virus (Matzeit *et al*., 1991; Ugaki *et al*., 1991) and maize streak virus (Palmer *et al*., 1999). These studies demonstrated that such vectors can tolerate very large inserts, in effect doubling the size of the geminivirus genome, without genetic instability being a serious issue. While an expression system based on deconstructed tomato yellow leaf curl virus (TYLCV) was capable of expressing GUS in monocots (Peretz *et al*., 2007), the use of geminiviral expression systems in monocots has generally remained limited.

Most of the efforts to develop viral expression vectors for monocots have focussed on the hordeivirus barley stripe mosaic virus (BSMV), a +ssRNA tripartite rod‐shaped virus. An early attempt to create a BSMV‐based vector for use in monocot cell cultures led the authors to the conclusion that the movement protein gene β2 (located on the RNA β) can be replaced with a GOI, but the coat protein gene β1 cannot be deleted, as this abolishes replication of RNA β by the proteins encoded on RNAs α and γ, even when a wild‐type RNA β is provided in *trans*. It transpired that, in fact, there is a *cis*‐acting element located just downstream of the coat protein gene that is required for efficient replication of RNA β (Petty and Jackson, 1990; Zhou and Jackson, 1996). Therefore, both β1 and β2 can be deleted without abolishing replication of RNA β, so long as the intergenic *cis*‐acting element is maintained, along with sufficient spacing between the ends of RNA β.

**Figure 6:**
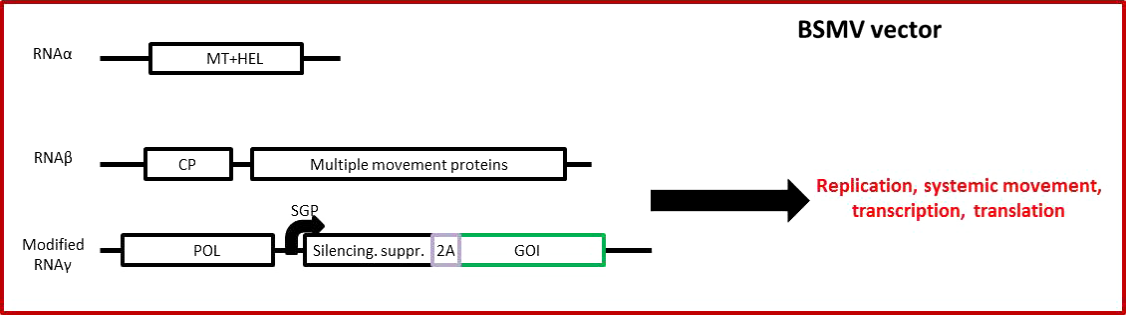
Simplified diagram of a BSMV‐based expression system. In this nondeconstructed system (Lee *et al*., 2012), the RNAα component supplies the viral methyl transferase (MT) and helicase (HEL) components, while RNAβ supplies the coat protein (CP) and multiple movement‐associated proteins. RNAγ contains the polymerase (POL) component and the γb silencing suppressor, which has been modified to contain the gene of interest (GOI) as a C‐terminal fusion via the self‐cleaving 2A peptide. The γb‐2A‐GOI fusion is under the control of a native viral subgenomic promoter (SGP). This expression system is capable of replication, cell‐to‐cell and systemic movement, transcription and translation.

The use of BSMV as a gene silencing and protein overexpression vector in whole barley plants is described by Lee *et al*. (2012). In this system, none of the viral genes are deleted, and the transgene is expressed through a genetic fusion of the transgene with the C‐terminus of the viral suppressor of silencing γ2 via a self‐cleaving 2A peptide (Figure 6). While this system works as an expression vector, it is severely limited by genetic instability. Because the CP is not necessary for viral replication and movement, an alternative may be to replace β2 with the GOI. However, this would also be problematic, because of the way modified BSMV is inoculated onto barley. This involves bulking up the three RNA components in *N. benthamiana* through agroinfiltration, then using (encapsidated) virus from this systemic infection to mechanically inoculate barley. This passage technique not only makes even small sequences inserted downstream of γ2 highly susceptible to deletion (Yuan *et al*., 2011), but also requires the presence of all of the viral proteins, including the CP and movement proteins. An alternative to the passage in *N. benthamiana* could be to inoculate barley with RNA prepared *in vitro*, but this is an impractical and inefficient process. This demonstrates the biggest hurdle facing transient expression in monocots: the impossibility to efficiently agroinfiltrate monocot leaves as is done with dicot leaves. This simple constraint prevents otherwise excellent expression vectors from being used efficiently in monocots, such as the potexvirus‐based FECT system described above (Liu and Kearney, 2010).

To overcome this hurdle, some research groups are attempting to improve the efficiency of agrobacterium‐mediated gene transfer in monocot leaves. Progress into this line of research has recently been demonstrated by Andrieu *et al*. (2012), who developed a protocol for the agroinfection of rice leaves for gene silencing purposes. They point out that inefficient agroinfection in cereal leaves is largely due to the physiological properties of the leaves. To address this, they wounded leaves from rice plants at tillering stage, then dipped the wounded leaves in a suspension of *Agrobacterium* containing a strong surfactant for up to an hour at 20 °C and then incubated the agroinfected plants at 20 °C for 2–3 days before returning them to a glasshouse. The authors show that the long agrobacterial incubation and the relatively low temperature promote bacterial binding to plant cells and increase agroinfection efficiency. While this is still not as efficient as agroinfiltration of dicot plants, it may prove sufficient to allow movement‐competent viral vectors to efficiently spread throughout monocot plants. If this technique were to be as successful in barley leaves as it is in rice leaves, it may allow the development of BSMV‐based vectors in which the coat protein gene is replaced by the GOI, and which can be directly agroinoculated onto barley leaves without prior passage in *N. benthamiana*. This, in turn, may alleviate the genetic instability issue seen with current versions of BSMV‐based vectors. Alternatively, improved agroinfection in monocots may allow the use of new or existing geminivirus‐ and potexvirus‐based vectors for gene silencing or protein overexpression. In the short term, developing high‐expression viral vectors for use in monocots would be advantageous for research into protein function. In the longer term, it may one day be useful to carry out molecular farming on a field scale using crop species for which agronomic practices are well established, although such a prospect raises some important regulatory and biocontainment issues.

## Conclusion

The past thirty years have seen plant molecular farming change from being of interest to only a few adherents, to becoming a cutting‐edge field of biotechnology, with plant‐produced proteins now beginning to reach the market as commercial products (Chen *et al*., 2013; Hefferon, 2012; Yusibov *et al*., 2011). This would certainly not be the case without the development of modern virus‐based overexpression vector systems, which were developed largely as a result of the synergy between plant viral vectors and agrobacterium‐mediated gene transfer. Deconstructed tobamovirus‐ and potexvirus‐based expression systems allow for very high levels of transgene expression, but their reliance on RNA replication makes the expression of large proteins less efficient and makes the co‐expression of multiple proteins from the same construct problematic. Geminivirus‐based expression systems can handle far larger inserts, but expression levels tend to peak early before gene silencing or Rep‐induced necrosis occurs. The CPMV‐*HT* system relies solely on translational efficiency as opposed to replication, while tobravirus‐based vectors are ideal for gene silencing and protein expression in roots. While this review has focussed on deconstructed vectors designed from the most commonly used viral taxons, it is important to note that there has been some work aimed at developing deconstructed vectors based other viruses, such as the cucumovirus cucumber mosaic virus (CMV; Fujiki *et al*., 2008) and the closterovirus beet yellows virus (BYV; Prokhnevsky *et al*., 2015).

Taken together, these findings suggest that future developments may focus on combining key elements of the different expression systems which are not mutually exclusive. For example, a P19 cassette with the CPMV‐*HT* UTRs could conceivably be combined with a geminivirus‐based LSL system to direct the efficient replication and hypertranslation of one or multiple GOIs from the same T‐DNA. Moreover, different strains or related species of different viruses could be studied with a view to optimize the individual components of different expression systems, such as the replicases and suppressors of silencing. Furthermore, the plasmid backbones of the different expression vectors may be optimized with a view to increasing ease of cloning as well as plasmid copy number in *Agrobacterium* cells, which may in turn result in greater transfection efficiency and higher yield of recombinant protein. The use of viral vectors may also be further developed for use in woody plants (Naylor *et al*., 2005) and monocots (Lee *et al*., 2012). Finally, it may be of use to the research community for comparisons of different expression systems to be carried out. Indeed, it is difficult to judge whether a yield reported with one expression system is limited intrinsically by the properties of that protein or by the expression system. Such comparison studies are few and far between (Dugdale *et al*., 2013; Larsen and Curtis, 2012; Liu and Kearney, 2010; Matić *et al*., 2012), and these have a tendency to compare two expression systems using a reporter protein under the conditions optimal for just one of the systems. For example, Dugdale *et al*. (2013) compare protein yield obtained with the INPACT expression system at 4 days postethanol induction with the yield obtained with pEAQ‐*HT* at 4 days postinfiltration (dpi), even though optimal yield for pEAQ‐*HT* is typically 6–7 dpi (Sainsbury *et al*., 2009). However, these studies do indicate that yields of recombinant proteins can differ significantly from those published elsewhere for the same protein using the same expression system, as in the case of Liu and Kearney (2010) comparing the FECT expression system with TRBO (Lindbo, 2007). It could therefore be of interest to see rigorous side‐by‐side comparisons of different expression systems for a wide range of different proteins, including single polypeptides as well as hetero‐oligomeric protein complexes.
